# Redox-proteomes of human NOS1-transduced versus MOCK SH-SY5Y neuroblastoma cells under full nutrition, serum-free starvation, and rapamycin treatment

**DOI:** 10.1016/j.dib.2018.10.078

**Published:** 2018-10-26

**Authors:** Juliana Heidler, Lucie Valek, Ilka Wittig, Irmgard Tegeder

**Affiliations:** a Institute of Clinical Pharmacology, Goethe-University Hospital, Frankfurt, Germany; b Functional Proteomics, Goethe-University, SFB815 Core Unit, Frankfurt, Germany

## Abstract

Upregulations of neuronal nitric oxide synthase (nNOS/NOS1) in the mouse brain upon aging and stress suggest a role of NO-dependent redox protein modifications for age-associated protein imbalances or dysfunctions. We generated a cell model, in which constitutive expression of nNOS in SH-SY5Y cells at a level comparable with mouse brain replicates the aging phenotype, that is, slowing of cell proliferation, cell enlargement, and expression of senescence markers. nNOS+ and MOCK cells were exposed to proteostasis stress by the treatment with rapamycin or serum-free starvation versus control conditions. To analyze NO-mediated S-nitrosylations (SNO) and other reversible protein modifications including disulfides and sulfoxides, we used complimentary proteomic approaches encompassing 2D-SNO-DIGE (differential gel electrophoresis), SNO-site identification (SNOSID), SNO Super-SILAC, SNO BIAM-Switch, and Redox-BIAM switch. The redox proteomes were analyzed using hybrid liquid chromatography/mass spectrometry (LC/MS). Full scan MS-data were acquired using Xcalibur, and raw mass spectra were analyzed using the proteomics software MaxQuant. The human reference proteome sets from uniprot were used as templates to identify peptides and proteins and quantify protein expression. The DiB data file contains MaxQuant output tables of the redox-modified proteins.The tables include peptide and protein identification, accession numbers, protein, and gene names, sequence coverage and quantification values of each sample. Differences in protein redox modifications in MOCK versus nNOS+ SH-SY5Y cells and interpretation of results are presented in (Valek et al., 2018).

Specifications table

**Table:** 

Subject area	*Redox Biology, Neuroscience, Proteomics*
More specific subject area	*Redox biology, Neurobiology, Aging, Protein homeostasis*
Type of data	*Spreadsheets*
How data was acquired	*Liquid chromatography / mass spectroscopy.* Thermo Scientific LTQ Orbitrap XL or Thermo Scientific™ Q Exactive Plus equipped with an ultra-high performance liquid chromatography unit and a Nanospray Flex Ion-Source
Data format	Excel Table 1: 2D-SNO DIGE
	Excel Table 2: SNOSID
	Excel Table 3: SNO-SILAC
	Excel Table 4: Gene ontology of redox modified proteins (pooled hits of 2D-DIGE, SNOSID and SNO-SILAC)
	Excel Table 5: SNO-BIAM switch
	Excel Table 6: Redox-BIAM switch
Experimental factors	*Cell treatment and harvest, protein extraction and labeling of redox sites, storage at -80°C, chromatography and MS analysis, MaxQuant software and Perseus software, gene ontology annotation*
Experimental features	*nNOS+ (neuronal nitric oxide synthase, NOS1) and MOCK transduced SH-SY5Y neuroblastoma cells with rapamycin versus vehicle treatment (time course, 6 h and 24 h) or serum-free starvation for 24 h versus full-nutrient conditions*
Data source location	*Frankfurt, Germany*
Data accessibility	*Data is with this article*

**Value of the data**

• The SNO and redox proteome datasets are useful to gain insight into nitric oxide mediated redox changes of protein homeostasis in the context of aging.
• The datasets provide information about redox modifications of proteins under nutrient stress evoked by rapamycin and starvation.
• The data reveal the usefulness and comparability of multiple approaches of redox proteomics and constitute a reference set for redox modifications in disease models.

## 1 Data

We performed a set of different redox proteome analyses in MOCK and nNOS+ SH-SY5Y cells to assess NO-mediated and other reversible redox modification including sulfoxidation and disulfide bridges in the context of deranged proteostasis evoked by treatment with rapamycin or serum-free starvation. Culture, transduction, and stimulations are described in [1].

The data are MaxQuant output files (Table 1 2D-SNO-DIGE, Table 2 SNOSID, Table 3 SNO-SILAC, Table 4 GO of SNO modified proteins, Table 5 SNO-BIAM switch, and Table 6 Redox-BIAM switch) providing essential information including peptide and protein identification, accession numbers, protein and gene names, sequence coverage, and quantification values of each sample. Identifications from the reverse decoy database, identified by site only and known contaminants were excluded. The Excel file also contains Gene Ontology (GO) terms associated with the SNOed proteins (Table 4 and specified columns in Tables 5 and 6). The GO overrepresentation analysis (Table 4) was performed with the Panther GO overrepresentation tool (http://www.pantherdb.org/).

## 2 Experimental design, materials, and methods

### 2.1 Cell culture and transduction

Briefly, SH-SY5Y were grown in RPMI 1640 medium supplemented with heat-inactivated 10% fetal bovine serum, 100 U/ml penicillin/streptomycin, and 2 mM glutamine at 37 °C, 5% CO_2_ in humidified atmosphere. For SILAC experiments (stable isotope labeling by amino acids in cell culture), cells were grown in SILAC™ Protein ID & Quantitation Medium (Invitrogen) containing SILAC stable isotopic [^13^C_6_]-L-arginine and [^13^C_6_] L-lysine and supplements as above for at least 10 passages.

A stable cell line of nNOS expressing SH-SY5Y cells was produced by lentiviral-mediated transduction of nitric oxide synthase 1 (nNOS/NOS1) using a bicistronic NOS1-IRES-EGFP expression vector (GeneCopoeia, Mm04153 pReceiver-Lv; IRES, internal ribosomal entry site). Control cells were transduced with the control lentiviral vector and are referred to as MOCK. Transduced cells were FACS sorted according to their GFP expression (FACS-Aria Cell sorter, Becton Dickinson).

To induce mTOR-dependent autophagy MOCK and nNOS+ cell were grown to 60%–70% confluence and were stimulated with 1 µM rapamycin for 0.5, 4, 8, and 24 h (2D-SNO-DIGE), or 6 h, or 24 h. An equal volume of vehicle (DMSO) was added to the control cells. During stimulation, cells were supplemented with NOS cofactors including 10 µM NAD, 40 µM NADPH, and 100 µM tetrahydrobiopterin. For starvation, cells were washed and cultured in serum-free medium for 24 h.

### 2.2 Redox proteomics principle

The principle of the analyses of posttranslational redox modifications in proteins is similar for all techniques, which were employed. The techniques evolved over time, which is revealed in the increasing number of protein hits obtained. The least sensitive is the 2D-SNO-DIGE because it requires protein separation by 2-dimensional gel electrophoresis, comparison of gels, spot picking, and subsequent analysis via mass spectrometry. Only strongly expressed protein can be analyzed with this technique. SNO-site identification (SNOSID) identifies the site of modification but does not provide quantitative data. The most sensitive and advanced methods are the SNO-BIAM and Redox-BIAM Switch assays. They provide quantitative data and identify the site of modification simultaneously. However, compared with SNOSID, fewer sites are identified. The SNO-ELISA is useful for confirmation of candidate proteins but does not provide "omics" data.

In the first step, non-oxidized sulfhydryl groups are masked with N-ethylmaleimide (NEM). Subsequently, originally oxidized residues are reduced, either mildly with ascorbic acid to reduce S-nitrosylated SH-groups (SNO sites) or generally with DTT to reduce reversible oxidations including sulfoxides and disulfides. Finally, the now newly generated SH-groups (i.e., the original oxidation sites) are labeled with biotinylated iodoacetamide or with Cy-dyes. Proteins are trypsinized, modified peptides are captured via streptavidin and analyzed per liquid chromatography/mass spectrometry or ELISA. The details of the different extraction procedures and LC/MS settings are described below.

### 2.3 2D-SNO DIGE

The SNO-DIGE relies on 2D separation of proteins by mass and isoelectric point, and was done as described using Cy-Dyes [2]. The experiment comprised five analytical gels from rapamycin-stimulated nNOS expressing SH-SY5Y cells at baseline and up to 24 h after adding rapamycin. SNO-DIGE identified key candidates, confirmed in subsequent assays.

Cell pellets were homogenized in Tris-Lysis buffer (7 M urea, 2 M thiourea, 4% Chaps, 30 mM Tris, 1:100 protease inhibitor cocktail (Roche), 100 μM neocuproine, pH 7.8) containing 25 mM N-ethyl-maleimide, NEM. Unsolubilized cell particles were sedimented for 20 min at 16,000 *x g* at room temperature. The supernatant was precipitated in ice-cold acetone overnight at −20 °C. Protein pellets were resolubilized in Tris lysis buffer and protein concentration was estimated by spectrometry (NanoVue Spectrophotometer; GE Healthcare). Analytical gels: Protein S-nitrosylation sites were reduced using 1 mM ascorbate and 10 µM CuSO_4_ in DIGE buffer (7 M urea, 2 M thiourea, 4% Chaps, 30 mM Tris, pH 7.8) and labeled for 1 h at room temperature with 4 nmol CyDye for scarce sample labeling (GE Healthcare, Freiburg, Germany) per 5 µg of protein. The samples were labeled with Cy5 and the pooled mixture was labeled with Cy3 to generate an internal standard. Each sample was mixed in a 1:1 ratio with internal standard together with equal amounts of rehydration buffer (7 M urea, 2 M thiourea, 2% (w/v) Chaps, 18 mM DTT, 0.5% IPG buffer pH 3–10 (GE Healthcare), 0.002% bromophenol blue). Proteins were separated by 2D isolelectric focusing (IEF)/SDS-PAGE. Preparative gel: Remaining lysates were pooled, reduced, and labeled with Cy3. 400 µg of total protein were loaded by passive rehydration overnight and separated by 2D IEF/SDS-PAGE and used for spot picking and protein identification by mass spectrometry.

The 2D SNO-DIGE analysis was composed of five analytical gels from rapamycin-stimulated nNOS expressing SH-SY5Y cells at baseline (time 0) and at 0.5 h, 4 h, 8 h, and 24 h after adding rapamycin. The pooled standard was used to normalize the spots. Gels were scanned with a Typhoon™ 9400 Imager (GE Healthcare) and analyzed by DeCyder™ 7.0 (GE Healthcare). The estimated number of spots in each map was set to 2500 and matched by automatic batch function. Match results were inspected and adapted by several rounds of setting landmarks and automatic rematching. Spots were compared across groups according to the standardized abundance, and proteins of interest were picked from the preparative gel using an Ettan™ Spot Picker (GE Healthcare) supplied with 2.0 mm picker head and transferred into perforated 96-well plates. The in gel digestion with trypsin and peptide extraction were done essentially as described [3]. Results are presented in Table 1.

### 2.4 S-nitrosylation site identification (SNOSID)

SNO-site identification (SNOSID) provides a qualitative assessment of SNO modified proteins and relies on sequential NEM masking and IAM labeling of trypsinized peptides, which are then analyzed by LC/MS and was done essentially as described [2], [4].

Ten microgram of protein from sample preparations were reduced with 1 mM ascorbate and 10 μM CuSO_4_ in trypsin digestion buffer (50 mM Tris, 1 mM CaCl_2_, pH 7.8) and labeled with 200 nM biotin-HPDP (Thermo Scientific). After 1 h incubation, 500 ng of MS grade trypsin (Promega) and 10% acetonitrile (Geyer) were added, and the solution was incubated at 37 °C for 16 h. Trypsin digestion was stopped with 0.5 mM phenylmethanesulfonylfluoride (PMSF) and the biotinylated peptides were purified with MyOne Streptavidin Dynabeads (Invitrogen) by incubation for 1 h. After washing with MS buffer (5 mM NH_4_HCO_3_, 10% acetonitrile, pH 8) the peptides were eluted with 5 mM DTT and incubated with 20 mM iodoacetamide for 30 min. Trypsin generated peptides of duplicate samples were then analyzed by mass spectrometry as described below. Results are shown in Table 2.

### 2.5 Biotin switch ELISA

Fifty microgram of protein were reduced with 1 mM ascorbate and 10 μM CuSO4 in TBS (50 mM Tris, 150 mM NaCl, pH 7.6) and labeled with 200 nM biotin-HPDP (Thermo Scientific). After 1 h incubation, the solution was precipitated in ice-cold acetone overnight at −80 °C. After overnight precipitation, the samples were centrifuged (16,000 x *g*, 4 °C, 20 min) and acetone was removed. The remaining pellet was allowed to dry for 30 min and then dissolved in 50 μl TBS followed by sandwich ELISA detection. Anti-Ube2d (ube2d, 1:1000, Abcam) was coated to the surface of a 96-well plate overnight at 4 °C in coating buffer (100 mM NaHCO_3_, 33 mM Na_2_CO_3_, pH 9.5). After washing, the plate was blocked with 1% BSA in PBS. Subsequently, 20 μg of the SNO site biotinylated proteins in blocking buffer were loaded per well and incubated overnight at 4 °C. After washing, anti-biotin IRdye-700 (1:500, manufacturer, Rockland, 600-130-098) was added for 2 h at room temperature and the infrared fluorescence was analyzed on an Odyssey Scanner. Gapdh was used as internal standard. Results are shown in [1].

### 2.6 Super SILAC analysis of protein abundance and SNO-modifications

The SNO-Super SILAC method employs standards of cells grown in SILAC medium supplemented with peptides to detect SNO-sites. The standards were split in two parts, one labeled with iodoacetamide (IAM) and the other with N-ethylmaleimide (NEM). The method allows for simultaneous quantification of S-nitrosylations and protein expression and the details of protein extraction and MS-analysis are described in DIB article "Full proteome". Results are presented in Table 3.

### 2.7 SNO-BIAM and redox-BIAM switch for SNO-modifications and reversible oxidations

The BIAM (EZ-LInk Iodoacetyl-PEG2-Biotin) switch assays were developed to identify redox-modified proteins with high sensitivity to allow for analysis of redox-modified pathways and protein networks.

MOCK and nNOS+ SH-SY5Y cells were washed with PBS containing 15 mM NEM and precipitated by the addition of 20% TCA and stored frozen at −20 °C. For the SNO-BIAM switch assay a trifluoroacetic acid (TCA)-aliquot was thawed on ice, centrifuged for 30 min with 16,000 x *g* and washed with 10% TCA and 5% TCA, respectively. Pellets were resuspended in 200 μl NEM-DAB (8 M Urea, 5 mM EDTA, 0.5% SDS, 100 µM Neocuproine, 50 mM Tris/HCL, pH 8.5, and 50x molar excess NEM) and incubated at 850 rpm for 1 h at 22 °C in the dark. Proteins were precipitated with ice-cold acetone, collected by centrifugation, washed, resuspended in 150 μl SNO-DAB (8 M Urea, 5 mM EDTA, 0.5% SDS, 50 mM Tris/HCL, pH 8.5, 10 µM CuSO_4_, and 1 mM ascorbate) and incubated at 850 rpm for 5 min at 22 °C in the dark. Subsequently, 150 μl BIAM-DAB (50x molar excess BIAM (EZ-link Iodoacetyl-PEG2-Biotin) in 8 M Urea, 5 mM EDTA, 0.5% SDS, 50 mM Tris/HCL, pH 8.5 were added followed by incubation at 850 rpm for 1 h at 22 °C in the dark. Proteins were precipitated with ice-cold acetone overnight at −20 °C, collected by centrifugation, washed, and resuspended in 100 μl lysis buffer (5 mM EDTA, 50 mM Tris/HCL pH 8.5, 1% Triton-X-100, 1% SDS). 200 µg of proteins were affinity purified using agarose streptavidin beads overnight at 4 °C on a wheel. After washing, beads were resuspended in 50 µl 6 M GdmCl, 50 mM Tris/HCl, pH 8.5 and incubated at 95 °C for 5 min. The samples were diluted with 25 mM Tris/HCl, pH 8.5, 10% acetonitrile to obtain a final GdmCl concentration of 0.6 M. Proteins were digested with 2 µg trypsin (sequencing grade, Promega) overnight at 37 °C under gentle agitation. Digestion was stopped by adding TCA to a final concentration of 0.5%. Peptides were loaded on multi-stop-and-go tips (StageTip) containing six C18 discs. Purification and elution of peptides was performed as described by Kulak and Mann [5]. Peptides were eluted in wells of microtiter plates, dried and resolved in 1% acetonitrile, 0.1% formic acid.

The REDOX-BIAM Switch assay was performed accordingly, first masking non-oxidized sulfhydryl groups with NEM, subsequent reduction with 3 mM DTT and then labeling of the newly generated SH-groups with BIAM as described above. The major difference between the assays relies in the harshness of the reduction process (i.e., ascorbic acid versus DTT).

### 2.8 Mass spectrometry and peptide/protein identification

Trypsinized peptides were dissolved in 5% acetonitrile supplied with 0.5% formic acid in water and separated on a 75 μm ID emitter tip (New Objectives) filled with ReproSil-Pur C18-AQ 120 °A, 3 µm (Dr. Maisch GmbH) and placed into the autosampler of the liquid chromatography unit (Agilent 1200 Nano-HPLC). Nano-HPLC runs (60 min for in-gel digests and 90 min for SNOSID and SILAC samples) were performed with an increasing acetonitrile (ACN) gradient from 5% to 50% containing 0.1% formic acid with a flow rate of 200 nl/min. Subsequently, the column was washed with 90% ACN for wash-out and re-equilibrated with 5% ACN, 0.1% formic acid. Eluted peptides were automatically submitted to ESI-MS/MS measurements by LTQ Orbitrap XL™ ETD (Thermo Scientific) for 2D-SNO-DIGE, SNOSID, and SNO_SILAC.

The MS data were acquired by a survey scan in a mass range of 400–1600 m/z followed by CID fragmentation of up to 5 precursor ions using Data Dependent Acquisition (exclusion time 3 min, rejection of singly charged precursor ions). Peak lists for SNO-DIGE were generated by Xtract_MSn (Thermo Scientific) and searched using Mascot Server (version 2.2) against a database containing human protein sequences downloaded from Uniprot. The Mascot search settings were as follows: Maximum missed cleavages 2, precursor mass tolerance 10 ppm, fragment ion tolerance 0.8 Da, and optional modifications allowed on methionine (oxidation) and cysteine (carbamidomethylation by iodoacetamide). Only peptides with individual ion scores indicating identity (*p *< 0.05) were considered.

SNOSID and SNO-SILAC were analyzed by MaxQuant (1.3.0.5) [6], [7] using the human uniprot database (August 2014, 68379 sequences). The MaxQuant settings were as follows: Maximum missed cleavages 2, main search precursor mass tolerance 5 ppm, fragment ion tolerance 0.5 Da, optional modifications allowed on methionine (oxidation), and cysteine (modification by NEM and carbamidomethylation by iodoacetamide) and acetylation at the N-terminus of proteins. FDR was set to 0.05. For SNOSID, the output table containing carbamidomethyl sites including peptide sequences and the posterior error probabilities (PEP) for each peptide was evaluated. Only SNO-sites where the best peptide PEP in the experiments was <0.015 were considered as candidates for S-nitrosylation (Table 2 for SNOSID and Table 3 for SNO-SILAC). Known contaminants and reverse hits were excluded from the lists. SILAC Light/Heavy pairs were used for quantification (Table 3). Sequence coverage and peptide spectra were analyzed by PEAKS7 (Bioinformatics Solutions Inc, Waterloo, Canada).

For the SNO-BIAM switch and Redox-BIAM-Switch, LC/MS analyses were performed on a Thermo Scientific™ Q Exactive Plus equipped with an ultra-high performance liquid chromatography unit (Thermo Scientific Dionex Ultimate 3000) and a Nanospray Flex Ion-Source (Thermo Scientific). Peptides were loaded on a C18 reversed-phase precolumn (Thermo Scientific) followed by separation on in-house packed picotip emitter tips (diameter 100 µm, 15 cm long from New Objectives) with 2.4 µm Reprosil C18 resin (Dr. Maisch GmbH). For SNO-BIAM-Switch, a gradient was used from mobile phase A (4% acetonitrile, 0.1% formic acid) to 40% mobile phase B (80% acetonitrile, 0.1% formic acid) for 60 min, and a second gradient to 80% B for 15 min with a flow rate 400 nl/min. For REDOX-BIAM Switch, the gradients were from mobile phase A (4% acetonitrile, 0.1% formic acid) to 30% mobile phase B (99% acetonitrile, 0.1% formic acid) for 90 min, and then to 60% B within 15 min, using a flow rate 400 nl/min. MS data were recorded by data dependent acquisition Top10 method selecting the most abundant precursor ions in positive mode for HCD fragmentation. Lock mass option [8] was enabled to ensure high mass accuracy between multiple runs. The full MS scan range was 300–2000 m/z with resolution of 70000, and an automatic gain control (AGC) value of 3 x 10^6^ total ion counts. The maximum ion injection time was 160 ms. Only higher charged ions (2+) were selected for MS/MS scans with a resolution of 17500, an isolation window of 2 m/z and an automatic gain control value set to 10^5^ ions. The maximal ion injection time was 150 ms. Selected ions were excluded if they occurred within a time window of 30 s (Redox-BIAM-Switch) or 20 s (SNO-BIAM) after a fragmentation event. Full scan data were acquired in profile mode and fragments in centroid mode by Xcalibur software.

Xcalibur raw files were analyzed using the proteomics software MaxQuant (1.5.2.8) [6] to identify peptides and proteins using the human uniprot reference proteome as template (Download April 2015, June 2017). The false discovery rate (FDR) was set to 1%. The data file includes peptide and protein identification, accession numbers, protein and gene names, sequence coverage, and label free quantification (LFQ) values of each sample. Identifications from the reverse decoy database, identified by site only and known contaminants were excluded.

MaxQuant data were further analyzed with ArrayStar (DNASTAR 15) and Perseus 1.6.0.2. [9] as described in the main manuscript.
